# Professionalism, stigma, and willingness to provide patient-centered safe abortion counseling and care: a mixed methods study of Ethiopian midwives

**DOI:** 10.1186/s12978-021-01238-0

**Published:** 2022-06-13

**Authors:** Addisu Fekadu, Aster Berhe, Belete Belgu, Ibrahim Yimer, Yeshitila Tesfaye, Sarah Jane Holcombe, Sahai Burrowes

**Affiliations:** 1 Ethiopian Midwives Association, Equatorial Guinea Road, Behind Elsa Kolo, Addis Ababa, Ethiopia; 2 UNFPA, Old ECA Building, 5th Floor, Menelik Avenue, Addis Ababa, Ethiopia; 3 Bill & Melinda Gates Institute for Population and Reproductive Health, Johns Hopkins School of Public Health, 615 N. Wolfe Street, Baltimore, MD 21205 USA; 4 Touro University, California Public Health Program, 1310 Club Drive, Vallejo, CA 94592 USA

**Keywords:** Ethiopia, Midwives, Abortion, Counseling, Quality, Patient-centered care

## Abstract

**Background:**

Midwives are a large proportion of Ethiopia’s health care workforce, and their attitudes and practices shape the quality of reproductive health care, including safe abortion care (SAC) services. This study examines how midwives’ conceptions of their professional roles and views on women who have abortions relate to their willingness to provide respectful SAC.

**Methods:**

This study uses a cross-sectional, mixed methods design to conduct a regionally representative survey of midwives in Ethiopia’s five largest regions (Oromia; Amhara; Southern Nations, Nationalities, and Peoples [SNNP]; Tigray; and Addis Ababa) with a multistage, cluster sampling design (n = 944). The study reports survey-weighted population estimates and the results of multivariate logistic regression analyzing factors associated with midwives’ willingness to provide SAC. Survey data were triangulated with results from seven focus group discussions (FGDs) held with midwives in the five study regions. Deductive and inductive codes were used to thematically analyze these data.

**Results:**

The study surveyed 960 respondents. An estimated half of midwives believed that providing SAC was a professional duty. Slightly more than half were willing to provide SAC. A belief in right of refusal was common: two-thirds of respondents said that midwives should be able to refuse SAC provision on moral or religious grounds. Modifiable factors positively associated with willingness to provide SAC were SAC training (AOR 4.02; 95% CI 2.60, 6.20), agreeing that SAC refusal risked women’s lives (AOR 1.69; 95% CI 1.20, 2.37), and viewing SAC provision as a professional duty (AOR 1.72; 95% CI 1.23, 2.39). In line with survey findings, a substantial number of FGD participants stated they had the right to refuse SAC. Responses to client scenarios revealed “directive counseling” to be common: many midwives indicated that they would actively attempt to persuade clients to act as they (the midwives) thought was best, rather than support clients in making their own decisions.

**Conclusion:**

Findings suggest a need for new guidelines to clarify procedures surrounding conscientious objection and refusal to provide SAC, as well as initiatives to equip midwives to provide rights-based, patient-centered counseling and avoid directive counseling.

**Supplementary Information:**

The online version contains supplementary material available at 10.1186/s12978-021-01238-0.

## Plain Language Summary

When health care workers refuse to provide safe abortion care (SAC) for religious, moral, or personal reasons, they jeopardize their clients’ health and violate the right to care. Scholars believe that health care workers’ professional commitments to patient care and to their profession’s goals can help them prioritize patient care over their personal biases. The Ethiopian government has assigned midwives a central responsibility to provide SAC, but there is no comprehensive understanding of Ethiopian midwives’ willingness to provide SAC and allied rationales, or the relationships between their sense of professional duty and willingness to provide.

To answer these questions, a survey and focus groups with midwives in Ethiopia’s five most populated regions were conducted. Almost half of midwives were unwilling to provide SAC, and half disbelieved that it was midwives’ duty to do so. Most believed that midwives should be able to refuse to provide SAC based on religious or moral objections. Midwives were motivated to provide care by a belief that clients would die without care and by a sense of professional duty. When asked about how they would treat women requesting abortion care and contraceptives, many midwives said that they would encourage the woman to do what the midwife him- or herself thought best, rather than support her in making her own decision.

These regionally representative findings suggest the need for new provider guidelines to clarify practices surrounding conscientious objection and refusal to provide safe abortion care and for programs to better train midwives to provide respectful counseling.

**Supplementary Information:**

The online version contains supplementary material available at 10.1186/s12978-021-01238-0.

## Background

Ethiopia is one of the few sub-Saharan African countries that have successfully expanded access to legal safe abortion care (SAC) services in the last 20 years [1]. In 2005, the country revised its criminal code to expand the conditions under which SAC could be provided legally. These conditions included cases of “rape or incest, a risk to the life or health of the mother, fetal malformation, and maternal disability or age and younger than 18 years” [2]. The reform authorized providers to accept women’s statements in cases of rape or incest without need for further validation when determining eligibility to receive services [3]. Since passage of Ethiopia’s reform, the proportion of abortions performed in health facilities has increased significantly, from approximately one-fourth of all abortions in 2008 to one-half in 2014 [4]. A key factor in the success of the reform was Ethiopia’s pioneering efforts to expand the number and cadres of practitioners authorized to provide SAC. Midwives’, nurses’, and health officers’ scopes of practice were broadened to include first-trimester abortion provision. Health officers with surgical training (“integrated emergency surgical officers”) were given the authority to provide second trimester abortion procedures [3]. Currently, midwives and similar advance practice professionals such as nurses and health officers provide more than 80% of abortions in Ethiopia [5]. Midwives and similar cadres of health professionals are increasingly globally recognized as qualified to provide such services [6, 7].

These task-sharing initiatives have taken place in a context of dramatic expansion of Ethiopia’s health workforce, a transformative national effort [8]. Midwives in Ethiopia have increased in number from 294 in 1984 to more than 16,000 in 2018 and are of two main types: bachelor’s degree midwives with four years of postsecondary training, and diploma midwives who have three years of specialized midwifery training [8]. The curriculum includes content on professional ethics and on safe abortion, although the actual practical training has varied by training institution [9–11]. As the health workforce has grown, and access to care has increased, Ethiopia’s Federal Ministry of Health has increasingly focused on improving the quality of care provided, emphasizing respectful, patient-centered care. This shift toward quality stems from the growing recognition that poor treatment of clients in the health sector violates human rights and deters the use of life-saving, facility-based care [12–15]. The need for measures of the quality of contraceptive and abortion care is recognized, but although some are under development, few have been institutionalized [16–19].

A particularly troubling aspect of low quality abortion care is practitioner refusal to provide SAC services for religious or moral reasons [20–22] or because of negative attitudes and stigma toward abortion. Practitioners who object to delivering SAC are often reluctant to make it clear that safe, legal services are available at their facility or to refer clients to other facilities [23, 24]. Refusal and conscientious objection have at times been found to be associated with tendencies toward directive, and at times coercive, counseling meant to persuade clients to continue pregnancy rather than to facilitate client decision making [25, 26].

Practitioner refusal to provide SAC adversely affects health. Refusal may cause delays in women obtaining care and can lead them to seek illegal, unsafe care outside of the formal health system [27]. Delays in care can, in turn, lead to greater risk of complications and reduced choice in the abortion care methods available [28, 29]. The negative impacts of refusal may be more severe in low-income settings where clients have less recourse to other practitioners [30] and among young, poor, and rural women for whom practitioners have been shown to be more likely to refuse care [31]. Individual practitioner refusal may contribute to systemwide gaps and interruptions in access to care in contexts like Ethiopia’s where access to care remains challenging. In 2016, in Ethiopia, fewer than half of all government facilities provided safe abortion services and adolescent/youth-friendly sexual and reproductive health services [32, 33].

Medical professionals refusing to offer life-saving but socially contentious reproductive health services on religious or other grounds is a global phenomenon [34, 35] but one that has not been well studied in Ethiopia. Prior research with midwives in Ethiopia suggests that despite high religiosity and moral misgivings, Ethiopian midwives have been more willing to provide SAC, and less likely to stigmatize clients seeking SAC, than other practitioners in sub-Saharan Africa [36–38]. However, overall rates of willingness are low and some stigmatizing attitudes are prevalent. Studies of provider refusal with regard to SAC in other sub-Saharan African countries have found that providers perceive abortion care as morally challenging and that refusal is common [39], both among those who profess to be conscientious objectors to providing care and those who do not [29]. In South Africa, the law on conscientious objection has been found to be poorly understood by practitioners, and conscientious objection there is largely unregulated [28, 40].

Professional guidelines [41, 42] and studies of refusal suggest that professional commitments to care and to the goals of a profession should lead practitioners to prioritize providing care over religious doctrines and should override particularistic biases [15, 38]. Expanding the understanding of professional responsibilities to include patient-centered care and the respect for patients’ rights is one way of promoting this commitment to quality care and ensuring continued access [15]. However, the integration of a patients’ rights framework into midwifery training is far from complete in Ethiopia. Professional ethics is included in pre-service training, but this training seems to narrowly focus on issues of confidentiality and privacy rather than on respecting rights and decision making [43]. Overall, it is not clear whether midwives view respecting rights as a core professional duty.

In Ethiopia, midwives are at the center of discussions about provider refusal and professional duties to uphold patients’ rights to SAC. They are often the first point of contact for clients, responsible for either providing SAC themselves or for referring clients requiring more complex care. Because midwives are a large and growing proportion of Ethiopia’s health care workforce, their attitudes and practices have the power to significantly improve (or compromise) the quality of SAC services and counseling nationally. There are also several other reasons to focus on midwives when studying practitioner refusal. First, midwives are a relatively new profession: formal midwifery training in Ethiopia started in 1954, and from 2008 to 2019, the country went from having fewer than 5 midwifery training institutions to having 48. This rapid educational expansion suggests that the norms of the profession may still be in flux and sensitive to input and policy. Moreover, because quality of care is often compromised during periods of rapid health sector expansion, keeping track of the beliefs and behaviors of this quickly growing cadre is crucial for ensuring the quality of reproductive health care services in Ethiopia [44]. Finally, research on practitioner opinion on abortion has shown that categories of practitioners requiring less formal education—e.g., nurses versus obstetricians/gynecologists—tend to be less supportive of abortion than their more educated counterparts [45–47]. This gap suggests that special attention should be paid to midwives’ attitudes and their impacts on quality of care.

Despite the centrality of midwives to ensuring access to high quality SAC in Ethiopia, relatively little is known about their attitudes about providing these services. This study seeks to help fill this gap by examining how midwives’ conception of their professional role with respect to SAC and how they view women who have abortions are related to their willingness to provide respectful SAC.

## Methods

This study used a cross-sectional, mixed methods design to collect two types of data. From October to December 2018, the Ethiopian Midwives Association (EMwA) collected quantitative data through a survey of 960 midwives at 408 health facilities, in 24 zones in Ethiopia’s five largest and most populous regions (Addis Ababa, Oromia, Amhara, Tigray, SNNP). In February–March 2019, EMwA also collected qualitative data through seven focus group discussions with midwives in the five study regions (Table 1).

**Table 1:** Characteristics of regional focus group discussions

Location	Number of participants	Type of midwife participant	Female	Male
Addis Ababa	6	Bachelor’s degree	4	2
Asela	6	Diploma	6	0
Hawassa	5	Diploma	5	0
Hosanna	8	Bachelor’s degree	1	7
Mekelle	6	Diploma	4	2
Nekemte	5	Bachelor’s degree	1	4
Shashemane	6	Diploma	5	1

### Multistage sample design

The study used a multistage, cluster sampling design to collect regionally representative data in order to produce findings that would be generalizable to public sector midwives in Ethiopia’s five most populous regions. Sampling began by selecting a sample of zones from the 68 administrative zones in Ethiopia’s five most populous regions for inclusion in the study. Originally, 30 zones were targeted for inclusion but because of logistical challenges caused by political unrest, only 24 zones in the five regions could be sampled. Zones were randomly selected by probability proportional to size based on the estimated number of midwives in the region where the zone was located.

**Fig. 1:**
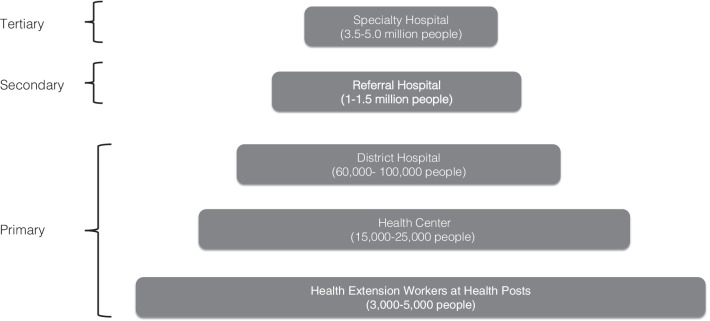
Ethiopia’s health system

For the second stage of sampling, within the selected zones, a Federal Ministry of Health (FMOH) list of all health facilities was stratified by facility type: tertiary/specialty hospital, referral hospital, district hospital, and health center (Fig. 1) to create a sampling frame [48]. All public non-specialty health centers and primary, secondary, and tertiary facilities were eligible for inclusion. Private facilities and specialty hospitals were excluded from the study.

Within each sampled zone, all tertiary hospitals were included in the study (i.e., there was purposive sampling with 100% probability of selection). Half of the referral and district hospitals in the zone were selected using simple random sampling from the aforementioned FMOH list. Health centers were selected using systematic sampling with probability proportional to size based on the estimated number of midwives in the zone.

The third stage of sampling occurred within health facilities using a convenience sampling strategy that differed by facility type. At health centers, all midwives on duty at the time of the study were surveyed (two per center). Three midwives were sampled from each primary and secondary hospital, and four midwives were sampled from each tertiary hospital on a first-come, first-served basis from hospital wards where SAC would normally be provided. All midwives who were permanent staff of the sampled facilities were eligible for inclusion in the study; volunteers and trainees were excluded.

### Sample size calculation

For the survey, the sample size was calculated to detect a 10% difference from past averages in the proportion of midwives who say that they are willing to provide SAC. Sample size calculations used a one-population proportion formula for cluster surveys with the assumptions that the proportion of practitioners willing to provide abortion services was 50%, based on previous Ethiopian studies [36–38]; a confidence interval of 95%; a power of 80%; a design effect of two that took into account two stages of sampling; and a 20% nonresponse rate. Using these assumptions, a sample size of 960 midwives was estimated (745 midwives adjusted for clustering and nonresponse).

### Survey instrument development

The survey instrument had been developed over two rounds of extensive cognitive and other testing in consultation with Ethiopian and international reproductive health experts (see Additional file 1). A detailed explanation of instrument development and validation can be found in a previous study [38]. The instrument was then translated into Amharic and Afaan-Oromoo and pre-tested in these languages.

The instrument contained questions on respondent demographics; work background and training; observations of disrespectful care; perspectives on SAC provision; readiness to provide services; and abortion-related stigma using seven items from the eight-item negative stereotyping subscale from Ipas’s Stigmatizing Attitudes, Beliefs and Actions Scale (SABAS) that had been validated with Ethiopian midwives [38, 49] (see Additional file 1). The majority of survey items were statements with possible responses on a five-point Likert scale of strongly disagree to strongly agree. Items of interest were then recoded to dichotomous variables with most items coded as agree or strongly agree equaling yes/high and unsure, disagree, or strongly disagree equaling no/low. The exception is the modified SABAS scale in which unsure responses were put in the high stigma category. This dichotomization strategy has been employed in prior studies using the SABAS instrument [38, 49].

### Survey data collection, quality, and analysis

EMwA selected three data supervisors and 30 data collectors from members of its regional chapters based on their past experience with data collection. All data collectors and supervisors participated in a three-day training, covering data management, survey administration, and survey contents and themes. Over two and a half months, from October 1 to December 20, 2018, data collectors traveled to each facility to recruit participants and hand deliver the paper survey instruments, which were self-administered, staying on hand to collect them the same day.

A dedicated data clerk entered survey data into an EpiInfo template. Data were then exported to Stata (version 16) for cleaning and analysis. In Stata, sampling weights were calculated based on the sampling design. All analyses were conducted in Stata using the svy prefix command to apply these weights.

Descriptive statistics by region are reported, along with the factors associated with midwives’ willingness to provide SAC, which were explored using multivariable logistic regression analysis. For the multivariable logistic regression, the main dichotomous outcome variable was willingness to provide SAC. Explanatory variables were selected based on a review of the literature [31, 37, 38]. They included sociodemographic factors such as age, marital status, and religious affiliation; professional characteristics such as SAC training and midwifery type; and attitudes towards SAC and people seeking care. Also included were dummy variables for the study regions, to control for potential unobserved regional factors that may be associated with willingness to provide care. The effect sizes of sociodemographic and attitudinal factors on willingness to provide SAC were reported in adjusted odds ratios (AOR), with their respective 95% confidence intervals (CI). Variables with estimated AORs that had *p*-values ≤ 0.05 were considered statistically significant.

### Focus group discussions

Survey data were triangulated with information from seven focus group discussions (FGDs) held with 42 midwives. Midwives were recruited from those attending regional EMwA in-service trainings. Training events were selected to ensure that there was an FGD in each of the five study regions and that there were at least two FGDs each for bachelor’s degree and diploma midwives. Within each training, convenience sampling was used to recruit midwives. All midwives attending training were eligible for inclusion in the study.

Discussions were held in the predominant local language at local training venues, using a guide developed by the research team that was modified iteratively as data were analyzed. The guides included three client care scenarios related to abortion and contraception that had been used in a previous study of Ethiopian midwives and respectful care [43] as well as questions on midwives’ attitudes towards SAC provision (see Box 1). Two leaders from EMwA, who had received training from a professional facilitator, moderated the discussions accompanied by a notetaker from EMwA.

FGDs were audio recorded with permission. Audio files were then simultaneously transcribed and translated into English by investigators. A team of six project investigators in Ethiopia and the United States hand coded transcripts using a codebook that contained a priori codes that were based on research questions, as well as codes that emerged from reviewing the data. Quotes that were illustrative of the themes in data were selected for reporting.

## Results

### Sample characteristics

The study surveyed 960 midwives (100% response rate), resulting in 944 usable surveys, and held focus group discussions with 42 midwives. Information on the population-weighted characteristics of the midwives in the study regions with their 95% confidence intervals can be found in Tables 2 and 3; see Table 1 for a description of the focus group participants.

**Table 2:** Population-weighted sociodemographic characteristics of midwives in Oromia, Amhara, SNNP, and Tigray regions and in Addis Ababa

	Number of observations^a^	Estimated percentage^b^	95% confidence interval^c^
Age categories			
Younger than 25 years	411	45	41, 49
26–40 years old	483	53	48, 57
Older than 41 years	21	3	1, 5
Sex			
Female	574	62	58, 66
Male	359	38	34, 42
Marital status			
Married	516	54	48, 60
Never married	397	44	37, 51
Widowed/divorced/separated	20	2	1, 5
Father’s education			
None	290	30	25, 36
Primary	273	29	25, 34
Secondary	138	16	13, 19
Higher	209	22	19, 26
Don’t know	14	2	1, 3
Have had children	392	43	37, 49
Have had unplanned pregnancy	295	27	23, 31
Currently using contraception	394	44	39, 48
Ethnic group			
Amhara	299	34	29, 39
Oromo	314	30	24, 36
Tigray	88	12	8, 18
Gurage	19	3	2, 4
Welayta	45	5	2, 12
Gamo	74	9	3, 20
Other	90	8	3, 20
Religious affiliation			
Ethiopian orthodox	536	62	55, 68
Muslim	130	13	8, 19
Evangelical or protestant	237	23	18, 29
Other	30	3	2, 4
Attendance at religious services			
Very regular (daily or > weekly)	383	42	39, 46
Regular (weekly)	237	23	20, 27
Less frequent (monthly, holy days, annually, never)	309	33	29, 38

^a^Unweighted observations. Because of weighting, the number of observations may not match percentages and may not add to the total sample size. Because of missing observations, proportions may not add to 100%

^b^These estimates are population weighted

^c^5% CIs reflect the range of possible values for the survey-weighted estimated percentages

**Table 3:** Population-weighted professional characteristics of midwives in Oromia, Amhara, SNNP, and Tigray regions and in Addis Ababa

	Number of observations^a^	Estimated percentage^b^	95% confidence interval^c^
Midwifery training program			
Diploma	509	54	49, 59
Bachelor’s degree	406	44	39, 50
Other	21	2	1, 3
Currently works in private facility	287	26	22, 31
Type of health care facility			
Rural health center	347	27	22, 33
Urban health center	311	41	36, 45
Primary hospital	126	15	12, 20
General hospital	117	14	11, 16
Specialized/tertiary/teaching	34	3	2, 6
Years in practice			
Fewer than 3 years	266	28	25, 32
3–4 years	244	29	26, 32
5–9 years	347	37	33, 41
10 years or more	53	6	4, 9
Has received SAC training	313	32	26, 39
Has had patient with incomplete abortion	748	85	81, 87
Has provided post-abortion care	620	72	66, 76
Has had patient die from unsafe abortion	143	17	15, 19
Has provided SAC	334	34	28, 42

^a^Unweighted observations. Because of missing observations and weighting, the number of observations may not match percentages and may not add to the total sample size

^b^These estimates are population weighted

^c^95% CIs reflect the range of possible values for the survey-weighted estimated percentages

### Willingness to provide safe abortion care

An estimated 54% (95% CI 45%, 63%) of midwives in Ethiopia’s five most populous regions reported that they were willing to provide SAC. A belief in the right to refuse to provide care was common: 60% (95% CI 56%, 64%) of midwives said that midwives should be able to refuse SAC provision on moral or religious grounds, and 50% (95% CI 44%, 55%) said that it was appropriate to refuse SAC services to an unaccompanied adolescent (Table 4).

**Table 4:** Population-weighted estimates of the proportion of affirmative responses to questions related to patient-centered SAC by region (n = 944)

	Addis Ababa	Amhara	Oromia	SNNP	Tigray	Total
Professionalism						
It is the professional duty of midwives to provide SAC *(agree or strongly agree)*	36% [35, 36]	51% [37, 64]	50% [40, 59]	48% [40, 56]	80% [67, 88]	50% [45, 55]
SAC access is good thing *(agree or strongly agree)*	66% [59, 72]	84% [79, 88]	72% [60, 81]	78% [70, 85]	89% [86, 91]	77% [72, 80]
SAC refusal risks mother’s life (*agree or strongly agree*)	46% [44, 47]	57% [45, 68]	54% [42, 65]	58% [50, 67]	81% [78, 83]	57% [52, 61]
Women will die without SAC (*agree or strongly agree*)	83% [74, 90]	77% [71, 82]	76% [63, 86]	82% [76, 87]	71% [59, 81]	79% [74, 83]
Midwives providing SAC make a positive contribution (*agree or strongly agree*)	66% [64, 68]	78% [71, 84]	71% [57, 82]	76% [69, 81]	93% [91, 95]	75% [71, 79]
Measures of respectful care						
Willing to provide SAC	38% [8, 82]	52% [41, 62]	60% [50, 70]	51% [40, 62]	79% [71, 85]	54% [45, 63]
Midwives should refuse adolescent asking for SAC (*agree or strongly agree)*	52% [42, 62]	66% [57, 74]	24% [16, 34]	61% [48, 72]	53% [36, 70]	50% [44, 55]
Midwives should be allowed SAC refusal (*agree or strongly agree)*	71% [69, 73]	58% [51, 64]	53% [41, 64]	65% [61, 69]	55% [46, 63]	60% [56, 64]
Attitudes towards women having abortion						
A woman who has had an intentional abortion cannot be trusted *(unsure, agree, or strongly agree)*^a^	40% [21, 61]	31% [23, 40]	29% [22, 37]	49% [39, 59]	34% [32, 36]	36% [31, 42]
A woman who has an abortion is committing a sin *(unsure, agree, or strongly agree)*^a^	59% [42, 75]	73% [69, 77]	41% [33, 50]	65% [33, 50]	42% [34, 51]	57% [52, 62]
Once a woman starts an intentional abortion, she will make it a habit *(unsure, agree, or strongly agree)*^a^	45% [45]	35% [29, 42]	31% [25, 37]	51% [46, 57]	35% [18, 58]	39% [37, 42]
A woman who has had an intentional abortion might encourage other women to do so *(unsure, agree, or strongly agree)*^a^	49% [40, 58]	48% [40, 55]	39% [30, 49]	61% [51, 70]	47% [40, 53]	49% [45, 53]
Not willing to provide because believe women make unjustified requests for SAC^b^	7% [2, 22]	14% [7, 24]	6% [3, 11]	12% [5, 27]	24% [9, 50]	11% [7, 17]
Not willing to provide because feel inadequately trained^b^	33% [14, 61]	25% [16, 37]	44% [34, 54]	40% [30, 51]	53% [52, 55]	38% [32, 46]
Not willing to provide because believe abortion is a sin^b^	54% [28, 78]	56% [40, 71]	32% [23, 43]	37% [31, 44]	13% [7, 22]	40% [32, 49]

95% CIs for survey-weighted estimated percentages reported in brackets

^a^These items are from the SABAS[49], an instrument with Likert scale response categories ranging from 1 (strongly disagree) to 5 (strongly agree). Because disagreement is considered as *less stigmatizing* for these SABAS questions, researchers are usually conservative in assigning ambiguous responses to the disagree category in order to avoid overstating the lack of stigma in respondents. Therefore, when this scale has been dichotomized in past studies, unsure responses have been categorized as affirmative as they do not indicate disagreement with stigmatizing statements [38, 58]. We have followed this convention

^b^n = 617

In discussions of the three client care scenarios (see Box 1), there was a mix of willingness and unwillingness to provide care to the women described in the scenarios. Reluctance to provide care was most pronounced in the scenario involving a married woman with children requesting safe abortion care (Scenario 2) and least pronounced in the scenario involving an adolescent girl seeking contraceptives.

The most common reason for being willing to provide SAC was a desire to help people avoid death or injury: 87% (95% CI 81%, 91%) of midwives who were willing to provide SAC cited this as their primary reason (data not reported in tables). The second most common reason for providing SAC was to help victims of forced sex, rape, or incest. This was cited by 9% (95% CI 7%, 13%) of those who were willing.

The survey results are supported by focus group findings in which the most common arguments that midwives gave for providing the services discussed in the scenarios were to avoid client suicide, injury, or death. Justifications based on avoiding client death and injury were mentioned in all seven focus groups. Even FGD participants who were unwilling to provide care acknowledged that the risk of self-harm and injury due to unsafe care was real and serious.

I will provide the service. Some of the women who have husbands and come for abortion services may change their mind with a little counseling, but others may not change their mind and they may even say “if you do not help me, I will hang myself or I will drink poison” …So, she may hang herself or drink poison if we do not provide services, which would lead her three children to be orphans. [Respondent H-4, diploma midwife]

Midwives’ concern about harm was most pronounced for the Scenario 1 involving the young girl requesting contraception (see Box 1). Here it was felt that the girl’s future health and career would be jeopardized if she had a child or sought unsafe care.

She has made up her mind [to have sex]; it could lead to worse problems if we don’t give her [contraception]: Problems to the family, the country, her own life… it could mess her up. [Respondent A-4, bachelor’s degree midwife]

In contrast, in Scenario 2, involving an older married woman with children seeking SAC (see Box 1), midwives frequently said that they would not provide the woman care because she should know about family planning and was responsible for her own pregnancy. In addition, in several focus groups, midwives stated that the woman should be able to handle an unplanned pregnancy because she already had children.

She has the capacity because she had three children. I will ask her the reason why she wants to abort and advise her not to abort and to have this baby. She can use long-term family planning to prevent further pregnancy. I would not give the service [Respondent H-3, diploma midwife]

First, with regards to her rights, she has to know her responsibilities. This is killing. She was supposed to use family planning services first. So, we will only give her advice. It is good to let her know that there are responsibilities just as there are rights. [Respondent S-2, diploma midwife]

Religious objections were the primary reason for being unwilling to provide care, cited by 40% (95% CI 32%, 49%) of unwilling midwives. The other frequently cited reasons for unwillingness were not feeling adequately trained to provide services (38% of those unwilling, 95% CI 31%, 46%) and believing that women make unjustified requests for SAC (11% of those unwilling, 95% CI 7%, 17%).

In contrast to the survey, most focus group participants did not cite religious objections consistently as their main reason for being unwilling to provide care. Strong religious objections were mentioned directly in four of the seven focus groups and by a minority of respondents within these groups. Midwives who had religious objections voiced them very strongly and expressed little flexibility in their refusal, except around providing post-abortion care services. However, many respondents were more nuanced in their responses, discussing circumstances under which they would and would not provide services. Several stated that their professional obligations and moral concerns for client safety overrode their religious convictions regarding abortion.

Every religion condemns abortion. In my religion, providing abortion is considered as taking someone’s life but it does not mean that the professionals who provide abortion services have no religion. I would regret if I refused to give service [to a patient] and she died while trying to abort using an unsafe procedure. It is our professional responsibility to do abortion. [Respondent H-4, diploma midwife]

Although religion was mentioned less often in focus groups than expected as a reason for refusal, moral arguments about what women’s ideal behavior should be permeated the discussions. In particular, there was a stated fear that providing services liberally would lead to people using them excessively. Midwives told stories of young girls using emergency contraception casually as birth control and of women having multiple abortions as reasons for interpreting the law on abortion care narrowly and restricting access.

Following the law is good, but in some cases if a woman is aborting her children repeatedly, it will have health consequences. The procedure should be done based on the law and education should be given for the mother. Otherwise, if anybody just does it repeatedly, this is not good. [Respondent S-6, diploma midwife]

I once met a 13 or 14-year-old girl who came to me…hmmm…she said, “I want HIV/AIDS testing”…[I said] “You are very young; have you had intercourse?” [She said] “Yes.” Then [I asked her], “Are you afraid of HIV/AIDS only or other related problems?” She was not afraid of any other problems. I mean she has used abortion and emergency contraception pills. They know lot of things, really a lot. I mean, she commonly used emergency contraception pills. I mean very well. So, this girl, you get what I am saying. [Respondent A-3, bachelor’s degree midwife]

Survey responses also indicate that a substantial proportion of midwives have negative views of abortion and of the people who have had them. An estimated 57% (95% CI 52%, 62%) of midwives saw abortion as a sin; 49% (95% CI 45%, 53%) thought women having an abortion would encourage others to have abortions; 40% (95% CI 37%, 42%) thought that women would make abortion a habit; and 36% thought that a woman who had an abortion could not be trusted (see Table 4). The overall mean score on the modified SABAS “negative stereotyping” subscale was 16 points out of a possible 35.

### Factors associated with willingness to provide SAC

In multivariable logistic regression, the factors significantly and positively associated with willingness to provide SAC were being male (AOR 1.55; 95% CI 1.02, 2.34), being widowed or divorced (AOR 4.31; 95% CI 1.19, 15.70), being a member of a small minority religious group (AOR 2.25; 95% CI 1.05, 4.81), believing that refusal risked clients’ lives (AOR 1.69; 95% CI 1.20, 2.37), believing that SAC was midwives’ professional duty (AOR 1.72; 95% CI 1.23, 2.39), and having been trained to provide SAC (AOR 4.02; 95% CI 2.60, 6.20) (Table 5). In contrast, being Evangelical Christian (AOR 0.69; 95% CI 0.48, 0.99) and believing that abortion was a sin (AOR 0.35; 95% CI 0.22, 0.54) were negatively associated with willingness to provide SAC. There was no significant association between geographic region and willingness to provide SAC in multivariable analysis.

**Table 5:** Factors associated with willingness to provide SAC, logistic regression (n = 816)

	AOR	95% CI
Age group (base = less than 25 years old)		
26–40 years old	1.03	0.63, 1.67
Older than 41 years	1.05	0.67, 1.66
Gender (base = female)		
Male gender	1.55*	**1.02, 2.34**
Marital status (base = married)		
Never married	0.88	0.54, 1.44
Widowed/divorced/separated	4.31*	**1.19, 15.69**
Have had children (base = no children)		
Have had children	1.10	0.66, 1.82
Religion (base = Ethiopian Orthodox)		
Muslim	1.21	0.77, 1.90
Evangelical Christian or Protestant	0.69^*^	**0.48, 0.99**
Other religious group	2.25^*^	**1.05, 4.81**
Religious attendance (base = very frequent religious attendance)		
Regular religious attendance	1.42	0.93, 2.16
Less frequent religious attendance	0.98	0.64, 1.49
Midwifery training program (base = diploma)		
Bachelor’s degree	0.79	0.53, 1.17
Training (base = no)		
Received SAC training	4.02^***^	2.60, 6.20
Attitudes towards women having abortion		
Belief that a woman who has an abortion is committing a sin	0.35^***^	**0.22, 0.54**
Belief that it is a professional duty to provide SAC	1.72^**^	**1.23, 2.39**
Belief that SAC refusal risks mother’s life	1.69^**^	**1.20, 2.37**
Region (base = Addis Ababa)		
Amhara	1.43	0.20, 10.41
Oromia	1.49	0.17, 13.38
SNNP	1.69	0.18, 15.71
Tigray	1.95	0.21, 17.79
Observations	**816**	

*p < 0.05

**p < 0.01

***p < 0.001

Because nonresponses to survey items led to 128 observations being dropped from the logistic regression model, we examined the robustness of our results by running an alternative model with a data set containing imputed values for missing responses. The magnitude and significance of associations remained similar in the new model with the exception of the religious affiliation variables, which faded in significance.

### Professionalism and willingness to provide SAC

Survey respondents were asked several questions about the importance of SAC and their professional role in providing SAC services. Almost 79% (95% CI 74%, 83%) of midwives reported that they felt women would die if SAC were not available, and three-quarters (75%, 95% CI 71%, 79%) reported believing that midwives providing SAC make a positive contribution to society. However, although a large majority (77%, 95% CI 72%, 80%) agreed that women having access to SAC was a good thing, only half (50%, 95% CI 45%, 55%) agreed that SAC was part of midwives’ professional duties. Although a few focus group participants did mention professional duty and patients’ rights as reasons for providing care, neither duty nor rights were consistently mentioned in discussions. Instead, a professional concern for client health was the dominant justification for providing care in these discussions.

### Secondary focus group themes

#### Directive counseling and education as alternatives to care

For those who refused to provide the care requested in the scenarios, the most common alternative care offered was intensive health education or counseling to convince women to change their behavior, and long detailed client histories taken in order to get to the “root of the problem.” This focus on getting to the root of problems was minor subtheme, present in all seven FGDs, that reflected a paternalistic sense among midwives that that it was their responsibility to solve client problems and to help mediate client conflicts by, for example, bringing in family members or spouses to discuss the client’s request for care.

As for me, what I am going to do is, I will try to convince her that she is not old enough for the contraceptive service and I will try to convince her to communicate with her parents. If she does not allow me to communicate with her parents, I will not give her the contraceptive [Respondent N-3, bachelor’s degree midwife]

Health education and counseling was rarely spoken about as a way to help women make informed decisions but rather as a means for convincing women to take a particular course of action. This was true even of midwives willing to provide care, who often said that they would initially counsel the client intensively and only if she persisted in her request would they provide care reluctantly.

First of all, I will try to counsel her in detail on the advantages and disadvantages. If she won’t accept the counseling I will give her the contraception because if not, she may have an unsafe abortion and she may die. [Respondent N-5, bachelor’s degree midwife]

I will make her change her mind by deep counseling. I will also tell her to take care for the future. [Respondent S-6, diploma midwife]

#### Law as justification for providing and refusing care

The law was used to justify both the provision and denial of services. Many midwives used ambiguities in the case scenarios to argue for strict interpretation of the law. The need to follow the law closely (as a professional duty) was used both to explain care refusals and to justify taking detailed client histories.

Except for causes stipulated in the abortion law of the country, I am not willing to provide abortion services. [Respondent A-1, bachelor’s degree midwife]

Interviewer: Which one [person] is right?

The person who follows the law properly and who aborts only in cases with complications, and when it is required, is right and should be encouraged. Otherwise, it [providing care] is causing harm. [Respondent S-2, diploma midwife]

In focus groups, the understanding of the law was muddy, with disagreements among participants about the specifics about how the law pertained to the scenarios. This was especially the case in Scenario 3 involving a woman with mental health problems seeking abortion care (see Box 1), where midwives were unclear whether mental illness was an acceptable condition for accessing care under the law (it is) and whether the mental illness had to be stabilized prior to providing care. They also disagreed on whether economic hardship was a stipulated condition for having an abortion under the law (it is not). Many of the midwives who said that they would not provide care to a woman seeking an abortion for reasons of economic hardship also said that if the woman’s husband accompanied her and agreed to the abortion, they would provide care even though a husband’s permission is not required by the law and does not alter the legality of providing services in this scenario.

I won’t do the abortion. Living in a rural area isn’t a reason to abort the child. I would ask whether she has discussed it with her husband and if they both decided it I might do it, but since she has a husband, I don’t think it is good to abort it, unless she has a breast-feeding child. [Respondent S-2, diploma midwife]

It was clear that whether they refused to provide care or not, many midwives felt that they had discretion to decide how strictly to interpret the law in practice. In almost every group discussion, there was at least one midwife, who for reasons of client safety, was willing to interpret the law loosely, including informing clients of the legal categories for abortion care so that clients could present themselves as belonging to one of the legally acceptable categories. Similarly, in every group there were midwives who said that they would refuse or delay care regardless of the legality of the request.

**Box 1:** Client care scenarios

Scenario 1: Woyzerit Miriam, an unmarried 14-year-old client, comes into your facility. She works as a housemaid in the town where this health facility is located. She asks for a contraceptive method. She also asks that you not tell her parents or other relatives or employers	
Scenario 2: Woyzero Tsehai, a 24-year-old married woman with three children, comes into your health center and requests that you help her by providing safe abortion care. The health center is far from any other health facility	
Scenario 3: Woyzero Selamawit, a 30-year-old unmarried woman is suffering from severe mental illness. She comes into your health center and requests that you help her by providing safe abortion care services	

### Need for external authorization

A second underlying theme highlighted by the Scenario 2 discussion of the pregnant woman in economic hardship (see Box 1), was one that runs through all of the discussions and scenarios, namely, a tendency for midwives to want to bring in external authority figures to approve women’s requests for care. In Scenario 1, midwives frequently said that they would attempt to bring in the young woman’s parents or employers to discuss her request for contraception; in Scenario 3, they said that they would contact the mentally ill woman’s family before providing care; and for Scenario 2, the majority said that they would ask the woman to bring in her husband to approve her request.

But no matter how much she wanted and insisted, unless she came with her husband, we would not provide her with the abortion. We may hear her problem. We may give an ear to hear whether her problem can be cause for abortion, finally we will tell her to come the next day, thinking deeply about it, and preferably with her husband. [Respondent M-5, bachelor’s degree midwife]

When discussing why they needed a husband’s authorization to perform the requested abortion in Scenario 2, respondents frequently mentioned a fear of legal reprisal or the husband’s violence. Fear of family reprisal was also given as a reason not to provide care in the other scenarios.

It makes you legally accountable, especially without any indications. If her husband comes in and asks, “Who killed my fourth child?” Who will be accountable for that? [Respondent N-5, bachelor’s degree midwife]

## Discussion

This study set out to examine Ethiopian midwives’ willingness to provide SAC and their understanding of their professional responsibilities to provide these services.

The study found moderately high rates of unwillingness to provide SAC in Ethiopia’s five most populous regions, with 46% of midwives stating that they were *not* willing to provide the service. This estimate is almost identical to that found in a 2013 study of the same population (44% unwilling) [37]. A 2016 study of Ethiopian midwives found higher rates of unwillingness (51%), but this may have been due to the study’s low response rates due to political unrest during the data collection period, which may have led to self-selection of conscientious objectors [38]. Together, these estimates present a relatively stable unwillingness rate of 44–52% suggesting that a large portion of Ethiopia’s midwives are unwilling to provide a life-saving and legal service.

What appears to be a clear binary split in the survey data between willing and unwilling practitioners becomes more nuanced when focus group discussions are examined. Here, rather than solid categories of objectors and providers, there are what other researchers have called “partial objectors” to SAC: practitioners who agree to provide care on a case-by-case basis using morally infused personal criteria to decide who is worthy of care [29, 50]. As in a recent study in Zambia, this study finds that refusal exists on a continuum with practitioners gauging the moral acceptability of providing care based on a shifting calculus of the reasons given for seeking care and the potential negative impact of not providing care [29].

Although most midwives expressed a commitment to following Ethiopia’s abortion law, they often seemed unfamiliar with the law’s requirements and demonstrated a willingness to impose personal criteria for deciding whether clients should be able to access services. Ethiopia’s law only permits abortion under a set list of conditions deemed necessary of protecting the health of clients and, as such, does not allow for conscientious objection. However, this lack of a formal conscientious objection exemption was not mentioned directly in any of the focus groups. Overall, there was a marked reliance among midwives on their personal judgment of the worthiness of clients when making clinical decisions and an idiosyncratic application of law to justify personal prejudices and views.

In addition to outright (hypothetical) refusal to provide care, responses to client care scenarios displayed large amounts of directive counseling in which client-provider communication focused on persuading a client to take a particular course of action. The growing consensus in the field of sexual and reproductive health is that ethical counseling focuses on providing information to help clients make their own informed decisions (informed choice) rather than encouraging people to make a particular decision (directive counseling) [51, 52]. Accordingly, in contraceptive and abortion care provision, providers’ priority should center on protecting the rights of clients, particularly their right to autonomy, and should avoid directive counseling [17, 53–55]. Our findings align with those from a recent study of Zambian health care practitioners where both those willing and those unwilling to provide SAC indicated that they would use counseling to delay care and/or change clients’ minds in order to get them to continue their pregnancies [29]. As in our study, willing practitioners in Zambia said that they would proceed with abortion care only if clients insisted.

To a surprising degree, midwives in our study said that they would rely on the input of external authority figures—husbands, parents, employers, and family members—to guide their decisions about whether to provide the requested care. This tendency has not been noted in similar studies, including in Ethiopia. It could be that calling on husbands and parents is a tactic to delay decision making or a way for focus group participants to avoid making a clear statement to researchers about their intentions. Their reference to external authority figures could also reflect midwives’ growing perceptions of, and a desire to deflect, public controversy over abortion in their care settings. Future studies of abortion care quality should examine whether this is indeed a common practitioner behavior in Ethiopia.

Looking at those who are willing to provide SAC, practitioners are often conflicted, viewing abortion care as problematic and morally fraught even as they agree to deliver such services. Fifty seven percent of midwives surveyed agreed, strongly agreed, or were unsure about the statement that “a woman who has an abortion is committing a sin,” including 28% of those who were willing to provide care. Practitioners also used moral and ethical arguments to justify provision of care, namely, the moral obligation to help people in need of life-saving services. These mixed, conflicting views have been reported in other African settings and seem to be the norm among practitioners providing these services in conditions of high maternal mortality and high abortion stigma [29, 38, 56, 57].

Many Ethiopian midwives have a public health rather than a rights-based rationale for providing SAC, with concern for the client’s health being by far the most common reason given for willingness to provide care in both the survey and focus group discussions. This public health focus among practitioners is in keeping with the framing used to advocate for and pass the penal code reform that liberalized the country’s law on abortion [1, 2, 11], and midwives’ lack of rights-based rationales may reflect a relative lack of focus on human rights in the training and advocacy for SAC in Ethiopia. Although a public health framing may have been an effective strategy for liberalizing legal access to safe abortion care, the high level of unwillingness to provide, even among those who know that abortion is lifesaving, highlights a potential weakness in relying solely on this framing.

Many of the factors found to be significantly associated with willingness to provide SAC in past Ethiopian studies are also significant here, namely, male gender (positive association), membership in evangelical Christian/Protestant churches, and stigma (negative associations) [37, 38], although it must be noted that the impact of religious affiliation was sensitive to model specification in our study. The factors with the strongest and most consistent positive association with willingness to provide care across different model specifications were having being trained to provide SAC, agreeing with the statement that SAC refusal risked women’s lives, and viewing the provision of care as a professional duty. Unlike previous studies, this study found no association between willingness to provide SAC and overall religiosity, as measured by religious service attendance. Focus group findings lend support to the survey results. For example, reflecting the significance of the “sin” variable in regressions, religious objections were mentioned by the discussion participants who were the most strident and unwavering in their opposition to SAC or contraceptive provision. However, the law, the characteristics of the client in the care scenario being discussed, and a lack of SAC training were just as often, if not more frequently, mentioned by participants as reasons for not providing care in focus groups. Although SAC training is an important factor in both the survey and focus group data, it is notable that in focus groups, some respondents said that they had refused training because they did not want to provide SAC. Therefore, it is not clear if the strong positive association between training and willingness to provide SAC reflects the positive impact of training or the fact that only those willing to provide SAC are trained. This finding deserves further study.

A sense of professional duty was one of the most reliable predictors of willingness to provide care in logistic regression models. In addition, in focus groups, several religious midwives noted that their professional obligations to save lives overrode their religious objections. However, only half of midwives agreed or strongly agreed that it was the professional duty of midwives to provide SAC, and two-thirds agreed or strongly agreed that midwives should be able to refuse providing care based on religious or moral objection. In line with the survey findings, many midwives hypothetically refused to provide care in the client care scenarios. In short, the study finds that midwives recognize the need for SAC provision to prevent morbidity and mortality but do not fully embrace it as a professional duty. Together, this and the previous findings outlined above suggest that for large segments of Ethiopians, whether or not they receive life-saving services will be dependent on the idiosyncratic evaluations of individual practitioners.

### Strengths and limitations

This study uses a rigorous research design to collect regionally representative quantitative data from the five regions containing 87% of Ethiopia’s midwives using validated, pre-tested instruments. The study triangulates survey findings with focus group data from midwives across the five study regions. Despite these strengths, the study has several limitations. First, although clustering of responses by health care facility was taken into account when making our sample size calculations, the multistage clustered design may have increased the size of the standard errors in our models, reducing the likelihood of observing significant associations between variables. This may explain why religiosity, which has been found to be significantly associated with willingness to provide SAC in previous studies [37], is not found to be consistently significant in this study. Dropped observations (n = 128) in the multivariable logistic regression due to nonresponses on survey items may have biased estimates if respondents included in the estimation sample differed significantly from those in the survey sample, although chi-squared tests reveal no statistically significant differences between the two samples except that those in the survey sample were more likely to be from Addis Ababa and less likely to see SAC provision as a professional duty. There were also limitations regarding precision in our qualitative data: difficulties with focus group translation quality may have led us to misinterpret some statements made in the discussions. In addition, as with any study of this kind, there is a possibility of recall and social acceptability biases in survey and focus group responses. Social acceptability biases may be present because data collection was carried out by members of the professional association to which respondents belong. However, the fact that respondents openly and candidly stated positions that were contrary to government and EMwA policy suggests that this risk was minor here.

Two limitations point to areas for future study: our survey lacked follow-up questions on views regarding abortion referrals and views of patients’ rights, which could have given us a more fine-grained view of midwives’ perspectives on their responsibilities with respect to SAC. Relatedly, because the study did not observe care or speak with clients, it could not gauge the quality of SAC provided.

## Conclusion

Our findings suggest three potential priorities for strengthening midwives’ ability to provide patient-centered counseling and reproductive health care in Ethiopia. The first recommendation is a renewed focus in supervision and training on patient-centered care, which could improve quality and provide an avenue for bolstering Ethiopian midwives’ professional commitment to providing SAC. The high prevalence of directive counseling found in this study suggests the need for organizational support to enable midwives to provide rights-based, patient-centered counseling. Pre- and in-service education can potentially better communicate professional expectations for midwives, particularly in supporting the rights and priorities of the client.

Second, good measures of the degree to which midwives and other medical professionals offer quality patient-centered care that supports the agency/autonomy of patients with respect to abortion are needed. Few measures of quality focusing on patient agency exist for either family planning or abortion care services [16].

Third, our finding that only half of midwives considered SAC provision a professional duty suggests that policy and training should clarify procedures and practices surrounding refusal to provide SAC. Ethiopia would be wise to heed the lessons learned in South Africa where “unregulated conscientious objection” has led to widespread confusion among practitioners about the parameters of refusal, the use of ad hoc, arbitrary decision making, and increases in unsafe abortions [28, 40].

## Supplementary Information

**Additional file 1:** Midwives and safe abortion care questionnaire

## Data Availability

Available upon request. Requests can be sent to the Ethiopian Midwives Association via Dr. Sahai Burrowes (sahai.burrowes@tu.edu).

## Abbreviations

AOR: Adjusted odds ratios
CI: Confidence interval
EMwA: Ethiopian Midwives Association
FGD: Focus group discussion
FMOH: Federal Ministry of Health
SABAS: Stigmatizing Attitudes, Beliefs and Actions Scale
SAC: Safe abortion care
SNNP: Southern Nations, Nationalities, and Peoples

## References

[1] Chavkin W, Stifani BM, Bridgman-Packer D, Greenberg JMS, Favier M (2018). Implementing and expanding safe abortion care: an international comparative case study of six countries. Int J Gynaecol Obstet.

[2] Bridgman-Packer D, Kidanemariam S (2018). The implementation of safe abortion services in Ethiopia. Int J Gynaecol Obstet.

[3] Federal Ministry of Health Ethiopia. Technical and Procedural Guidelines for Safe Abortion Services in Ethiopia 2nd edn. Addis Ababa, Ethiopia: Ministry of Health, FDRE; 2014.

[4] Guttmacher Institute. Induced Abortion and Postabortion Care in Ethiopia [Internet]. 2017 Jan. https://www.guttmacher.org/sites/default/files/factsheet/ethiopia_fact_sheet_final.pdf

[5] Gebrehiwot, Fetters, Gebreselassie, Moore, Hailemariam, Dibaba, et al. Changes in morbidity and abortion care in Ethiopia after legal reform: national results from 2008 and 2014. Int Perspect Sex Reprod Health. 2016 10.1363/42e1916 PMC5568644 28825903

[6] Berer M (2009). Provision of abortion by mid-level providers: international policy, practice and perspectives. Bull World Health Organ.

[7] World Health Organization. Health worker roles in providing safe abortion care and post-abortion contraception [Internet]. Geneva: World Health Organization; 2015 p. 81. http://www.who.int/reproductivehealth/publications/unsafe_abortion/abortion-task-shifting/en/26401543

[8] Ethiopian Midwives Association. State of Ethiopia’s Midwifery. Addis Ababa, Ethiopia; 2019.

[9] Gelagay M, Ethiopian Midwives Association. Expanding and Strengthening Midwifery Workforce in Ethiopia: Achievements, Lessons Learned, and Way Forward [Internet]. Jhpiego; 2018. http://resources.jhpiego.org/system/files/resources/Learning%20Brief%20Midwifery%20FINAL%2003%2025%2019.pdf

[10] Faye S, Baily R, Admassu A, Adamu Y. The Nursing Education Partnership Initiative (NEPI) in the Federal Democratic Republic of Ethiopia: Cost Analysis of the Nursing and Midwifery Education Programs at University of Gondar College of Medicine and Health Sciences and Arbaminch College of Health Sciences. USAID Capacity Plus; 2015

[11] Holcombe SJ (2018). Medical society engagement in contentious policy reform: the Ethiopian Society for Obstetricians and Gynecologists (ESOG) and Ethiopia’s 2005 reform of its Penal Code on abortion. Health Policy Plan.

[12] World Health Organization. Prevention and elimination of disrespect and abuse during childbirth [Internet]. World Health Organization; 2014. http://www.who.int/reproductivehealth/topics/maternal_perinatal/statement-childbirth/en/

[13] Miller S, Abalos E, Chamillard M, Ciapponi A, Colaci D, Comandé D (2016). Beyond too little, too late and too much, too soon: a pathway towards evidence-based, respectful maternity care worldwide. Lancet Lond Engl.

[14] Koblinsky M, Moyer CA, Calvert C, Campbell J, Campbell OMR, Feigl AB (2016). Quality maternity care for every woman, everywhere: a call to action. Lancet Lond Engl.

[15] Glenton C, Sorhaindo AM, Ganatra B, Lewin S. Implementation considerations when expanding health worker roles to include safe abortion care: a five-country case study synthesis. BMC Public Health [Internet]. 2017 [cited 2020 May 1];17. https://www.ncbi.nlm.nih.gov/pmc/articles/PMC5609023/10.1186/s12889-017-4764-z PMC5609023 28934942

[16] Tumlinson K. Measuring quality of care: A review of previously used methodologies and indicators. New York: Population Council; p. 29. Report No.: Working Paper Two.

[17] Holt K, Dehlendorf C, Langer A (2017). Defining quality in contraceptive counseling to improve measurement of individuals’ experiences and enable service delivery improvement. Contraception.

[18] Darney BG, Powell B, Andersen K, Baum SE, Blanchard K, Gerdts C, et al. Quality of care and abortion: beyond safety. BMJ Sex Reprod Health. 2018 10.1136/bmjsrh-2018-200060 PMC6225511 29972364

[19] Dennis A, Blanchard K, Bessenaar T (2017). Identifying indicators for quality abortion care: a systematic literature review. J Fam Plann Reprod Health Care.

[20] Dickens BM, Cook RJ (2000). The scope and limits of conscientious objection. Int J Gynaecol Obstet.

[21] Diniz D (2011). Conscientious objection and abortion: rights and duties of public sector physicians. Rev Saude Publica.

[22] Faúndes A, Duarte GA, Osis MJD (2013). Conscientious objection or fear of social stigma and unawareness of ethical obligations. Int J Gynaecol Obstet.

[23] Figo Committee for the Ethical Aspects of Human Reproduction and Women’s Health null, International Federation of Gynecology and Obstetrics. Ethical guidelines on conscientious objection in training. Int J Gynaecol Obstet. 2015;128:89–90. 10.1016/j.ijgo.2014.10.005 25458410

[24] Coppola F, Briozzo L, Nozar F, Fiol V, Greif D (2016). Conscientious objection as a barrier for implementing voluntary termination of pregnancy in Uruguay: gynecologists’ attitudes and behavior. Int J Gynaecol Obstet.

[25] Keogh LA, Gillam L, Bismark M, McNamee K, Webster A, Bayly C (2019). Conscientious objection to abortion, the law and its implementation in Victoria, Australia: perspectives of abortion service providers. BMC Med Ethics.

[26] Rowlands S (2014). Abortion care.

[27] Van Bogaert LJ (2002). The limits of conscientious objection to abortion in the developing world. Dev World Bioeth.

[28] Harries J, Cooper D, Strebel A, Colvin CJ (2014). Conscientious objection and its impact on abortion service provision in South Africa: a qualitative study. Reprod Health.

[29] Freeman E, Coast E (1982). Conscientious objection to abortion: Zambian healthcare practitioners’ beliefs and practices. Soc Sci Med.

[30] Chavkin W, Leitman L, Polin K (2013). Conscientious objection and refusal to provide reproductive healthcare: a White Paper examining prevalence, health consequences, and policy responses. Int J Gynecol Obstet.

[31] Morrell KM, Chavkin W (2015). Conscientious objection to abortion and reproductive healthcare: a review of recent literature and implications for adolescents. Curr Opin Obstet Gynecol.

[32] Gebrehiwot Y, Liabsuetrakul T (2009). Trends of abortion complications in a transition of abortion law revisions in Ethiopia. J Public Health.

[33] Moore AM, Gebrehiwot Y, Fetters T, Wado YD, Bankole A, Singh S, et al. The estimated incidence of induced abortion in Ethiopia, 2014: changes in the provision of services since 2008. Int Perspect Sex Reprod Health. 2016 10.1363/42e1816 PMC5568682 28825902

[34] Rehnström Loi U, Gemzell-Danielsson K, Faxelid E, Klingberg-Allvin M (2015). Health care providers’ perceptions of and attitudes towards induced abortions in sub-Saharan Africa and Southeast Asia: a systematic literature review of qualitative and quantitative data. BMC Public Health.

[35] Ngwena C (2003). Conscientious objection and legal abortion in South Africa: delineating the parameters. J Juridical Sci.

[36] Abdi J, Gebremariam M (2011). Health providers’ perception towards safe abortion service at selected health facilities in Addis Ababa. Afr J Reprod Health.

[37] Holcombe SJ, Berhe A, Cherie A (2015). Personal beliefs and professional responsibilities: Ethiopian midwives’ attitudes toward providing abortion services after legal reform. Stud Fam Plann.

[38] Holcombe SJ, Burrowes S, Hailu D, Scott R, Berhe A (2018). Professional pragmatism and abortion stigma: assessing the performance of the Stigmatizing Attitudes, Beliefs and Actions Scale (SABAS) among Ethiopian midwives. Afr J Reprod Health.

[39] Awoonor-Williams JK, Baffoe P, Ayivor PK, Fofie C, Desai S, Chavkin W (2018). Prevalence of conscientious objection to legal abortion among clinicians in northern Ghana. Int J Gynaecol Obstet.

[40] Trueman KA, Magwentshu M (2013). Abortion in a progressive legal environment: the need for vigilance in protecting and promoting access to safe abortion services in South Africa. Am J Public Health.

[41] FIGO. Ethical Guidelines on Conscientious Objection. International Federation of Gynecology and Obstetrics; 2012 p. 26–7. 10.1016/j.ijgo.2014.10.005 25458410

[42] The African Commission on Human and Peoples’ Rights (African Commission), General Comment No 2 on Article 14 (1) (a), (b), (c) and (f) and Article 14 (2) (a) and (c) of the Maputo Protocol, at para. 26.

[43] Burrowes S, Holcombe SJ, Jara D, Carter D, Smith K (2017). Midwives’ and patients’ perspectives on disrespect and abuse during labor and delivery care in Ethiopia: a qualitative study. BMC Pregnancy Childbirth.

[44] Kruk ME, Gage AD, Arsenault C, Jordan K, Leslie HH, Roder-DeWan S (2018). High-quality health systems in the Sustainable Development Goals era: time for a revolution. Lancet Glob Health.

[45] Hammarstedt M, Jacobsson L (2005). Views of midwives and gynecologists on legal abortion. Acta Obstet Gynecol Scand.

[46] Warenius L, Faxelid E, Chishimba P, Musandu J, Ongany A, Nissen E (2006). Nurse-midwives’ attitudes towards adolescent sexual and reproductive health needs in Kenya and Zambia. Reprod Health Matters.

[47] Solo J, Festin M. Provider bias in family planning services: a review of its meaning and manifestations. Glob Health Sci Pract. 2019;GHSP-D-19-00130. 10.9745/GHSP-D-19-00130 PMC6816811 31515240

[48] Federal Ministry of Health Ethiopia. Master Health Facility Registry. nd.

[49] Shellenberg KM, Hessini L, Levandowski BA (2014). Developing a scale to measure stigmatizing attitudes and beliefs about women who have abortions: results from Ghana and Zambia. Women Health.

[50] Loi UR, Otieno B, Oguttu M, Gemzell-Danielsson K, Klingberg-Allvin M, Faxelid E (2019). Abortion and contraceptive use stigma: a cross-sectional study of attitudes and beliefs in secondary school students in western Kenya. Sex Reprod Health Matters.

[51] Fink LR, Stanhope KK, Rochat RW, Bernal OA (2016). “The fetus is my patient, too”: attitudes toward abortion and referral among physician conscientious objectors in Bogotá, Colombia. Int Perspect Sex Reprod Health.

[52] Moskowitz E, Jennings B (1996). Directive counseling on long-acting contraception. Am J Public Health.

[53] Dehlendorf C, Krajewski C, Borrero S (2014). Contraceptive counseling: best practices to ensure quality communication and enable effective contraceptive use. Clin Obstet Gynecol.

[54] Cavallaro FL, Benova L, Owolabi OO, Ali M. A systematic review of the effectiveness of counselling strategies for modern contraceptive methods: what works and what doesn’t? BMJ Sex Reprod Health. 2019 10.1136/bmjsrh-2019-200377 PMC7569400 31826883

[55] Hardee K, Kumar J, Newman K, Bakamjian L, Harris S, Rodríguez M (2014). Voluntary, human rights-based family planning: a conceptual framework. Stud Fam Plann.

[56] World Health Organization. Ensuring human rights in the provision of contraceptive information and services [Internet]. Geneva: World Health Organization; 2014 p. 26. http://www.who.int/reproductivehealth/publications/family_planning/human-rights-contraception/en/24696891

[57] Oppong-Darko P, Amponsa-Achiano K, Darj E. “I Am Ready and Willing to Provide the Service … Though My Religion Frowns on Abortion”—Ghanaian Midwives’ Mixed Attitudes to Abortion Services: A Qualitative Study. Int J Environ Res Public Health [Internet]. 2017 [cited 2019 Sep 23];14. https://www.ncbi.nlm.nih.gov/pmc/articles/PMC5750919/10.3390/ijerph14121501 PMC5750919 29207521

[58] Håkansson M, Oguttu M, Gemzell-Danielsson K, Makenzius M. Human rights versus societal norms: a mixed methods study among healthcare providers on social stigma related to adolescent abortion and contraceptive use in Kisumu, Kenya. BMJ Glob Health [Internet]. 2018 [cited 2019 Nov 18];3. https://gh.bmj.com/content/3/2/e000608 10.1136/bmjgh-2017-000608 PMC5841529 29527357

