# Supplementary material for: Professionalism, stigma, and willingness to provide patient-centered safe abortion counseling and care: a mixed methods study of Ethiopian midwives
Source: Reprod Health. 2022 Jun 13;19(Suppl 1):197. doi: 10.1186/s12978-021-01238-0 (PMC9195199; doi:10.1186/s12978-021-01238-0)

**MIDWIVES AND Safe Abortion CARE Questionnaire**

Survey Number: Institution Code (*survey administration location*)

Survey Date: Surveyor Code:

The Ethiopian Midwives Association (EMwA) is conducting this survey on midwives’ perspectives and experience related to abortion and other stigmatized reproductive health services in order to improve the quality of training and services. We thank you for the help you give us by answering the following questions.

**PART 1: Demographic Background**

**Please respond to the questions below by either circling response options or filling in the blank.**

| **Questions** | **Answers** |
| --- | --- |
| 1. How old are you? | _______ years old |
| 1. What is your gender? | 0= Female  1= Male |
| 1. What is your marital status?   *Please circle only one answer:* | 1=Married  2=Never married  3=Widowed  4=Divorced/Separated |
| 1. Do you have any children? | 0= No  1= Yes |
| 1. What is the highest level of school your father attended: none, primary, secondary, or higher?   *Please select only one answer:* | 1 = None  2 = Primary  3 = Secondary  4 = Higher  98 = Don't Know |
| 1. What is your religion?   *Please select only one answer* | 1 = Ethiopian Orthodox  2 = Muslim  3 = Evangelical Christian or Protestant  4 = Catholic  5 = Other, specify __________________  6 = None |
| 1. Apart from weddings and funerals, about how often do you attend religious services these days?   *Please select only one answer:* | 1 = Every day  2 = More than once a week (But not every day)  3 = Once a week  4 = Once a month  5 = Only on special holy days  6 = Once a year  7 = Less often  8 = Never, practically never |
| 1. What is your ethnic group? | 1 = Afar  2 = Amhara  3 = Guragie  4 = Hadiya  5 = Nuwer  6 = Oromo  7 = Sidamo  8 = Somali  9 = Tigray  10 = Welaita  11 = Other, specify: _________________ |
| 1. At any point, have you or your spouse or partner ever had an unplanned pregnancy? | 0 = No  1 = Yes |
| 1. Are you or your current spouse or partner currently using contraception? | 0 = No  1 = Yes |

**PART 2: WORK BACKGROUND, TRAINING, AND EXPERIENCE**

| **Questions** | **Answers** |
| --- | --- |
| 1. What is the name of the midwifery or other health professional training institution that you most recently attended? | ________________________ (Institution name) |
| 1. What is your profession?   *Please select only one option:* | 1 = Midwife  2 = Other, specify: __________________ |
| 1. How many years have you been a practicing health care provider? | ________ years |
| 1. Are you currently providing clinical care? | 0 = No  1 = Yes |
| 1. What is your highest level of education you have completed in your professional career?   *Please circle all that apply:* | 1 = Midwife (Diploma)  4 = Midwife (Bachelor’s degree)  5 = Midwife (Accelerated)  6 = Nurse Midwife  7 = Midwife (Master’s degree)  8 = Other, specify ________________ |
| 1. Please identify the main type of facility that you work in.   *Please circle only one answer:* | 1 = Rural Health Center  2 = Urban Health Center  3 = Primary Hospital  4 = General Hospital  5 = Specialized Hospital  6 = Other, specify _________________ |
| 1. Do you also work in a private health facility? | 0 = No  1 = Yes |
| 1. Have you ever received training to provide safe abortion care (SAC) services?   *(If yes, answer question* ***19****, if no, skip to question* ***21****)* | 0 = No  1 = Yes |
| 1. When did you receive this training on safe abortion care (SAC)? | 0 = pre-service training  1 = in-service training |
| 1. What did this training on safe abortion care include?   *Please circle all that apply* | 1 = Didactic lectures  2 = Practical clinical training on medical abortion  3 = Practical clinical training on manual vacuum aspiration (MVA)  4 = Other ______________________________ |
| **In the public health facilities where you have worked or trained:** | |
| 1. Has anyone (any client) ever asked you for information about how to terminate an unwanted pregnancy or about abortion services? | 0 = No  1= Yes |
| 1. Have you ever had a patient with an incomplete abortion? | 0 = No  1 = Yes |
| 1. Have you ever provided post abortion care (PAC) services? | 0 = No  1 = Yes |
| 1. In a health facility where you have worked or trained, have you ever heard of a patient who has died due to an unsafe abortion? | 0 = No  1 = Yes |
| 1. Have you ever provided safe abortion care services (SAC)? | 0 = No  1 = Yes |

**PART 3: SABAS instrument**

The following scale was developed by Ipas to measure people’s attitudes and perspectives related to abortion at the individual and community levels. Your responses will help us judge its applicability for Ethiopia and for providers. There is no “right” or “wrong” answer to these questions, so please base your responses on your **own** personal experience, knowledge, and views or opinions. We want to learn **your** perspectives.

Please put an “X” next to each of the following statements to indicate how much you agree or disagree with the statement. Please **respond to all 8 statements below**, and do not leave any blank.

| **Statements** | **Strongly Disagree** | **Disagree** | **Unsure** | **Agree** | **Strongly Agree** |
| --- | --- | --- | --- | --- | --- |
| 1. A woman who has an abortion is committing a sin. |  |  |  |  |  |
| 1. Once a woman has one abortion, she will make it a habit. |  |  |  |  |  |
| 1. A woman who has had an intentional abortion cannot be trusted. |  |  |  |  |  |
| 1. A woman who has an intentional abortion brings shame to her family. |  |  |  |  |  |
| 1. A woman who has had an intentional abortion might encourage other women to get abortions. |  |  |  |  |  |
| 1. A woman who has an intentional abortion is a bad mother. |  |  |  |  |  |
| 1. A woman who has an intentional abortion brings shame to her community. |  |  |  |  |  |
| 1. A woman who has an abortion should be treated differently from everyone else. |  |  |  |  |  |

**PART 4: provision of services**

**In your work or clinical training, please mark how frequently you have observed any of the following types of behavior by medical professionals during the past year (12 months).**

| **I have observed:** | **Never** | **Once or twice** | **Several times** | **Most of the time** |
| --- | --- | --- | --- | --- |
| 1. **Verbal abuse**, such as using harsh tones or shouting, using undignified language or threats, if patients don’t cooperate |  |  |  |  |
| 1. **Physical abuse,** such as slapping, pinching, or hitting a patient |  |  |  |  |
| 1. **Procedures conducted without consent**, such as not explaining to patient about procedures provided |  |  |  |  |
| 1. **Confidentiality violated**, such as giving medical results to the patient when others could hear |  |  |  |  |
| 1. **Discrimination based on mother’s age, marital status, ethnic group, or economic status**, such as worse treatment of younger or unmarried patients |  |  |  |  |
| 1. **Right to privacy not respected**, such as when a women is not covered when she is moved to the delivery room or not covered after delivery |  |  |  |  |
| 1. A midwife providing poorer quality care to a woman seeking safe abortion care services than to other patients. |  |  |  |  |
| 1. A midwife shouting at or scolding a woman seeking safe abortion care services. |  |  |  |  |
| 1. A midwife ignoring the physical pain of a woman seeking safe abortion care services. |  |  |  |  |
| 1. A midwife **not** checking the condition of her/his patient in the unit/ward because she had come for safe abortion care services. |  |  |  |  |
| 1. Women seeking safe abortion care services who wait until last to get care due to a midwife. |  |  |  |  |

**PART 5: Attitudes Toward Abortion and its provision as a Health Service**

Please put an “X” next to each of the following statements to indicate how much you agree or disagree with the statement.

| **Statements** | **Strongly Disagree** | **Disagree** | **Unsure** | **Agree** | **Strongly Agree** |
| --- | --- | --- | --- | --- | --- |
| 1. Midwives who provide safe abortion care services are making a positive contribution to society. |  |  |  |  |  |
| 1. The community does not value health professionals who provide safe abortion care services. |  |  |  |  |  |
| 1. If there are not safe abortion care services, too many women will die of unsafe self-induced abortions. |  |  |  |  |  |
| 1. Health care providers who provide safe abortion services deserve respect for the work that they do. |  |  |  |  |  |
| 1. Health care providers should be allowed to refuse to provide any procedure for which they have a moral or religious disagreement. |  |  |  |  |  |
| 1. If an adolescent comes alone to a health facility for safe abortion care services, a midwife should ask her to go home and return with a parent. |  |  |  |  |  |
| 1. Midwives who provide safe abortion care services help prevent maternal mortality and morbidity. |  |  |  |  |  |
| 1. Mass media (television, radio and newspapers) take a balanced view about health care providers offering safe abortion care services. |  |  |  |  |  |
| 1. If a midwife refuses to provide safe abortion care services, she or he is risking the patient’s life. |  |  |  |  |  |
| 1. Other health care providers question the technical skills of healthcare providers who provide safe abortion care services. |  |  |  |  |  |
| **Statements** | **Strongly Disagree** | **Disagree** | **Unsure** | **Agree** | **Strongly Agree** |
| 1. People question the ethics of healthcare providers who offer safe abortion care services. |  |  |  |  |  |
| 1. Other health care providers question the ethics of health providers who provide safe abortion care services. |  |  |  |  |  |
| 1. It is the professional duty of a midwife to provide safe abortion services. |  |  |  |  |  |
| 1. Health providers who offer safe abortion services would not want their neighbors to know this. |  |  |  |  |  |
| 1. It’s a good thing that women can obtain safe abortion care services. |  |  |  |  |  |

**PART 6: Your Perspectives on YOUR own willingness to provide LEGAL ABORTION services**

| 1. I feel adequately trained to provide safe abortion care services. | 1 = Strong Disagree  2 = Disagree  3 = Unsure  4 = Agree  5 = Agree strongly |
| --- | --- |
| 1. If I had received the necessary training, I would be willing to provide safe abortion care services. | 1 = Strong Disagree  2 = Disagree  3 = Unsure  4 = Agree  5 = Agree strongly |
| 1. I am willing to provide safe abortion care services in the facility where I work.   *If ‘Agree Strongly’ or ‘Agree’,* ***go to*** ***question*** **63***. Otherwise,* ***go to question* 67***.)* | 1 = Strong Disagree  2 = Disagree  3 = Unsure  4 = Agree  5 = Agree strongly |
| 1. If you are willing to provide safe abortion care services, what are the most important reasons?   *Please circle as many reasons as apply.* | 1 = Desire to help women avoid death or injury from unsafe abortions in Ethiopia  2 = Desire to help women who have been the victims of forced sex, rape or incest  3 = Desire to see only wanted children brought into the world  4 = Desire to provide comprehensive care for patients  5 = Desire to help pregnant students to continue their education and improve their economic future  6 = Belief that poor and rural women deserve the same access to services as women with more money  7 = Belief that women have the right to have access to safe abortion care services  8 = Other (please explain) ___________________ |
| 1. I can talk openly with my family about the fact that I have provided safe abortion care services. | 1 = Strong Disagree  2 = Disagree  3 = Unsure  4 = Agree  5 = Agree strongly |
| 1. I can talk openly with my friends about the fact that I have provided safe abortion care services. | 1 = Strong Disagree  2 = Disagree  3 = Unsure  4 = Agree  5 = Agree strongly |
| 1. I avoid telling people that I have provided safe abortion care services. | 1 = Strong Disagree  2 = Disagree  3 = Unsure  4 = Agree  5 = Agree strongly |
| 1. If you are NOT willing to provide safe abortion care services, what are the reasons?   *Please circle as many reasons as apply.* | 1 = I have not been adequately trained.  2 = I believe that abortion is a sin.  3 = My colleagues will give me a hard time if I provide safe abortion care.  4 = I believe that women make unjustified requests for safe abortion services.  5 = I prefer to provide other health care services.  6 = Other (please explain) __________________  _______________________________________  00 = I am willing to provide safe abortion care services. |
| 1. What are the biggest challenges that you face in providing abortion care services, **OR**, if you do not provide abortion care services, that other providers face in providing abortion care services?   *Select all that apply.*   - *For respondents who have not provided abortion care services, please select the challenges that you think apply to providers who do provide abortion care services.* | 1 = Inadequate training to offer abortion care services  2 = Lack of supplies or equipment  3 = Uncertainty about the law on abortion  4 = Lack of support from administration of health facility  5 = Lack of support from colleagues  6 = Moral or religious objections from others  7 = Internal moral or religious uncertainties  8 = Other (please explain):  _______________________________________ |

**Go to next page.**

1. Please share your thoughts below on the professional responsibilities of midwives, and the issues of providing safe abortion care services, maternal mortality prevention, and patients’ sexual and reproductive rights.

**----------------STOP HERE--------------------**

**Thank you** for taking the time to complete this questionnaire and contributing to strengthening the quality of midwifery. If you feel that you need to talk to someone about your experiences in either providing or receiving abortion care services, please contact the Ethiopian Midwives Association at 0116 18 92 75 oR 0116 18 30 67 to inquire about support.

**Data Collector’s observations**

*(to be filled in after all surveys has been completed by the midwife*

*and collected by the data collector)*


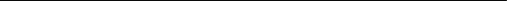

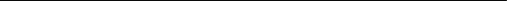

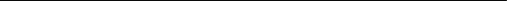

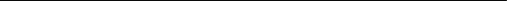

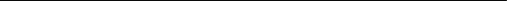

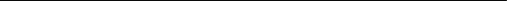

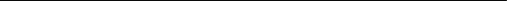

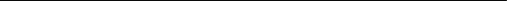

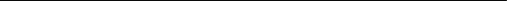

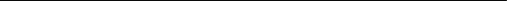

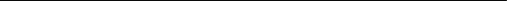

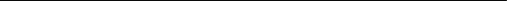

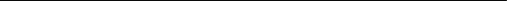


**SUPERVISOR'S OBSERVATIONS**


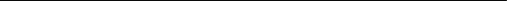

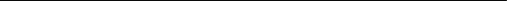

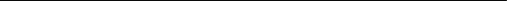

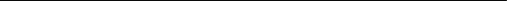

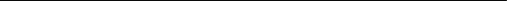

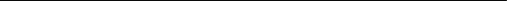

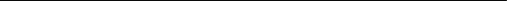

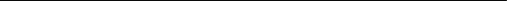

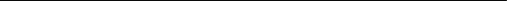

Supplement: Supplementary file 1 — Additional file 1: Midwives and safe abortion care questionnaire [file 12978_2021_1238_MOESM1_ESM.docx]
